# Bilateral oncoplastic breast-conserving surgery with volume replacement technique using the omental flap: a case report

**DOI:** 10.1186/s40792-022-01434-5

**Published:** 2022-05-09

**Authors:** Hisamitsu Zaha, Norie Abe, Hirofumi Matsumoto, Ayako Koki, Mikiko Unesoko

**Affiliations:** 1 Department of Breast Surgery, Nakagami Hospital, 610 Noborikawa, Okinawa, Japan; 2 Department of Pathology, Nakagami Hospital, 610 Noborikawa, Okinawa, Japan

**Keywords:** Breast-conserving surgery, Omental flap, Oncoplastic breast-conserving surgery, Volume replacement

## Abstract

**Background:**

Many oncoplastic volume replacement techniques have been reported, however, it is generally difficult to utilize a single distant flap for bilateral breast carcinomas.

**Case presentation:**

We report a case of bilateral multiple breast carcinomas successfully treated with immediate volume replacement technique with an omental flap. Bilateral partial mastectomies were performed for bilateral breast carcinomas (one in the left breast and two in the right breast). The pedicled omental flap was laparoscopically harvested, and divided at the mid-portion of the flap. The proximal half of the flap was used to fill the right defect, and the distal half of the flap filled two defects in the left breast. Cosmetic outcome was excellent with minimal donor-site scars.

**Conclusions:**

The omental flap can be considered for highly selected patients with bilateral breast carcinomas.

## Background

Oncoplastic breast-conserving techniques can be classified as volume displacement or volume replacement, and many local and distant flaps have been used for volume replacement immediately after partial mastectomy [1, 2]. However, it is generally difficult to utilize a single distal flap for bilateral breast carcinomas.

Herein, we report a case of bilateral multiple breast carcinomas successfully treated with a single laparoscopically harvested omental flap (OF).

## Case presentation

A 52-year-old lady was detected abnormal calcifications with segmental distribution by mammography in her left lower medial quadrant in October 2019. Ultrasound and MRI also found two small contralateral breast tumors which were approximately 5 mm in size and located in the right upper outer and the right lower medial quadrants. Core needle biopsies revealed a ductal carcinoma in situ in her left breast, an invasive ductal carcinoma in her right medial quadrant and an atypical ductal hyperplasia in her right outer quadrant. The patient had no family history of breast and ovarian carcinoma, and had no BRCA mutations on genetic testing. The patient insisted on preserving her breasts although her breast size was small. Then, bilateral breast-conserving surgery (BCS) with volume replacement technique using the OF was planned.

**Fig. 1:**
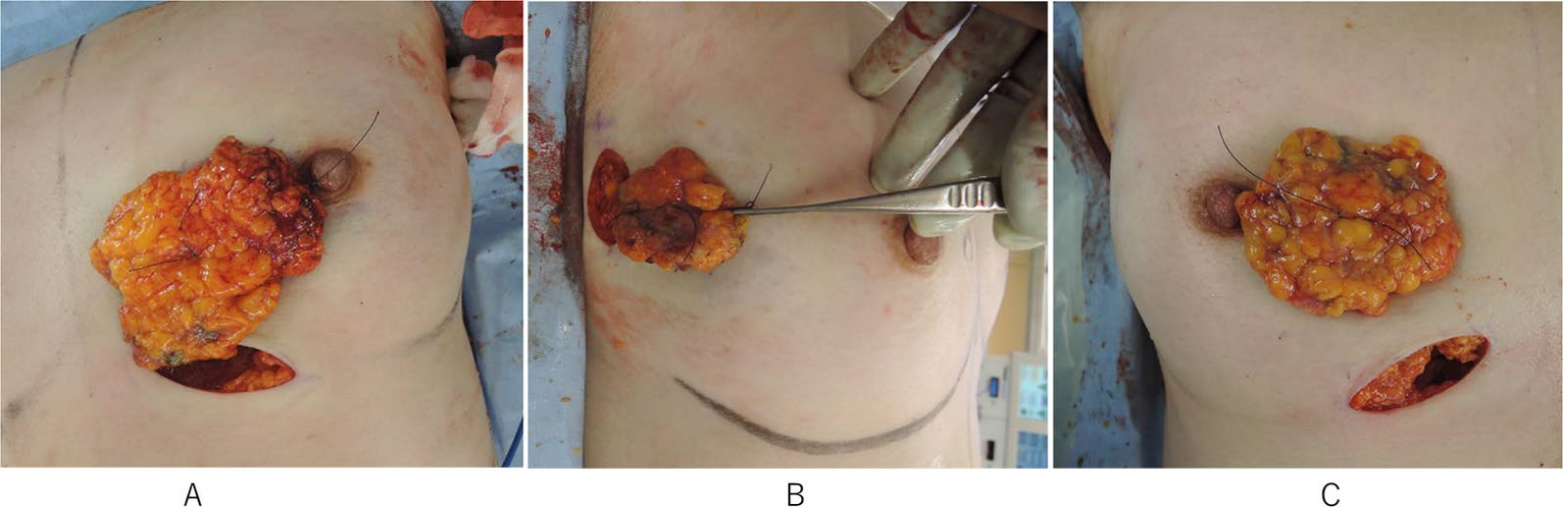
**A** Partial mastectomy with an inframammary fold incision for a tumor in the left lower medial quadrant. **B** Partial mastectomy with an axillary incision for a tumor in the right outer quadrant. **C** Partial mastectomy with an inframammary fold incision for a tumor in the right medial incision

Bilateral BCS was simultaneously performed with laparoscopic harvesting of the OF. First, left sentinel lymph-node biopsy (SNB) was carried out through a small axillary incision, then, a 5-cm-long skin incision was made along the left medial inframammary fold (IMF). A skin flap was created around the tumor and partial mastectomy was carried out (Fig. 1A). For the right tumors, a 4-cm-long axillary incision was used for SNB and resection of the tumor in the outer quadrant (Fig. 1B), and a separate 4-cm-long incision along the right medial IMF incision was used for the tumor in the medial quadrant (Fig. 1C).

**Fig. 2:**
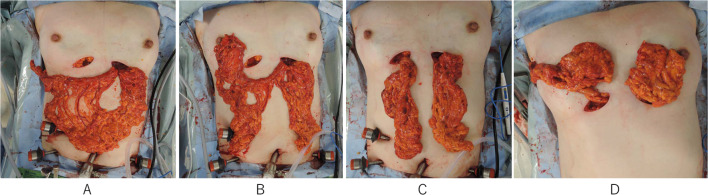
**A** The omental flap was extracted from the abdominal cavity through a subcutaneous tunnel. **B** The omental flap was divided between the 3rd- and 4th‐descending epiploic arteries preserving the main truck of the gastroepiploic vessels. **C** The proximal half of the flap was used to fill the defect in the left medial quadrant. ** D** The distal half of the flap was used to fill the defects in the right defects

The OF was laparoscopically harvested, as described in detail elsewhere [3]. After harvesting the OF, a subcutaneous tunnel was prepared toward the xiphoid process from bilateral IMF incision. When the tunnel reached the white line, a 2-finger-wide longitudinal incision is made to communicate with the abdominal cavity, and the OF was extracted from the abdominal cavity (Fig. 2A). The OF divided between the 3rd- and 4th-descending epiploic arteries preserving the main trunk of the gastroepiploic vessels (Fig. 2B, C). The proximal half of the flap was used to fill the defects in the right medial and outer quadrants passing through a subglandular tunnel (Fig. 2D).

**Fig. 3:**
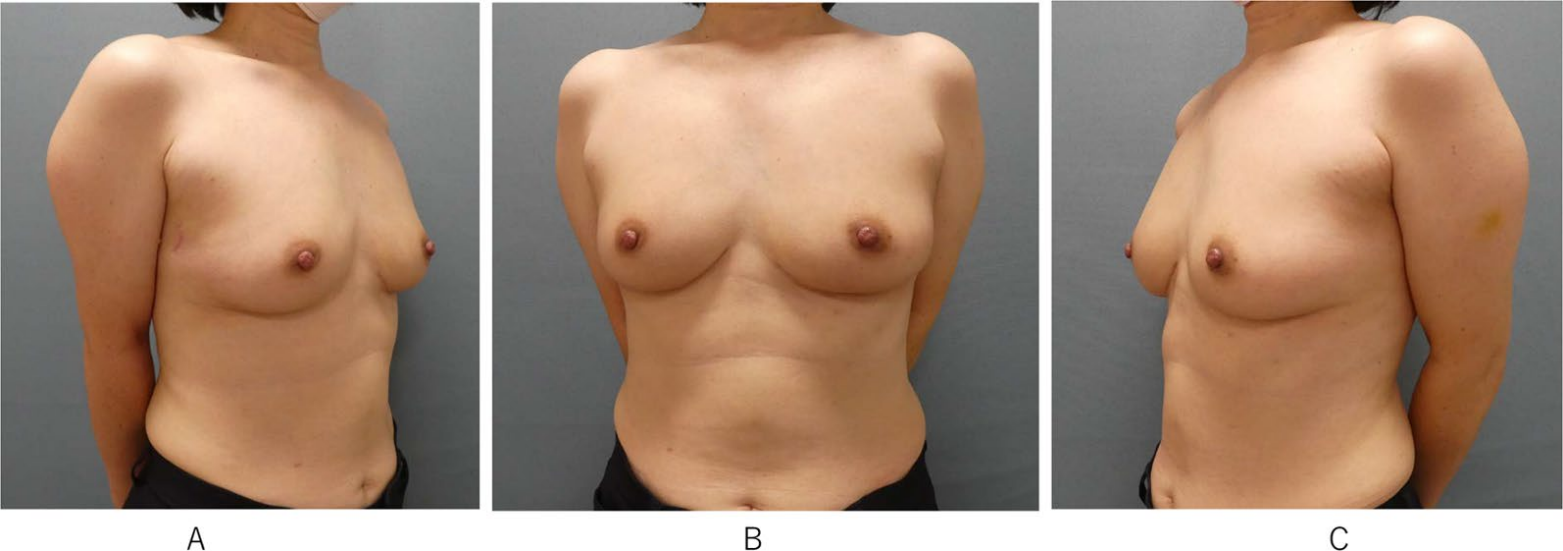
Post-operative pictures one year after surgery. **A** Right oblique view showed a small axillary scar. **B** Frontal view showed minimal breast and abdominal scars. **C** Left oblique view also showed excellent cosmetic outcome

Pathological findings revealed ① ductal carcinoma in situ in the left medial quadrant sized 25 × 8 × 7 mm; ② invasive carcinoma in the right medial quadrant sized 5 × 5 × 3 mm with ductal carcinoma in situ component sized 25 × 5 × 3 mm, and ③ ductal carcinoma in situ in the right outer quadrant sized 18 mm. All of the tumor margins were negative. Tumor characteristic of the right invasive carcinoma was ER-positive and HER2-positive. Then the patient underwent post-operative adjuvant systemic therapy with four cycles of TC (docetaxel plus cyclophosphamide) concurrent with trastuzumab for one year and subsequent bilateral radiation therapy and endocrine therapy. Post-operative course was uneventful and cosmetic result was excellent one year after surgery (Fig. 3).

## Discussion

Oncoplastic BCS consists of volume displacement and volume replacement techniques. A lot of procedures have been reported as volume replacement techniques [4–9]. Each technique has advantages and disadvantages, and has suitable quadrants to utilize. Thus, the surgeon must make a correct decision to choose an individualized and appropriate technique.

Both of an indication and a contraindication of the OF for breast reconstruction are described elsewhere [3, 5]. In our 200-case series of the OF, the successful rate of laparoscopically harvesting of the OF was 99.5%. The rate of complications including minor fat necrosis was 12.0%. Laparoscopy-associated complications occurred only in 4 cases (2.0%). One major vascular injury to the main trunk of the gastroepiploic artery fell into total loss of the OF. Late complications include two ventral hernias in the infra-xiphoid area [5]. In brief, the OF flap has big advantages with its minimal donor-site scar and applicability to any quadrants because of its long pedicle. A disadvantage is inability to estimate preoperative volume of the flap [3, 5]. In the present case, the OF was divided and utilized for bilateral volume replacement. To our knowledge, it is the first case in which a single flap could replace bilateral partial mastectomy defects. A vascular anatomy of the OF is unique, in which the main gastroepiploic vessels supply several descending epiploic vessels. Thus, a blood supply of the flap is rich enough even the flap is divided at the middle part as long as the main gastroepiploic vessels are preserved. However, there are limitations of the OF for bilateral application. One is tumor locations. When the bilateral tumors are located in both the upper outer quadrants, it is difficult for the proximal half of the flap to reach the defect. At least, one of tumors should be located in the lower medial quadrant. The other is the volume of the defects which should not exceed 100 g each.

There would be concern about indication of bilateral BCS for three tumors. Negative for BRCA mutation cannot always exclude hereditary breast cancer. Synchronous bilateral breast carcinomas are relatively rare, and the choice of BCS is not well studied. The study from Sloan Kettering Cancer Center reported that patients with synchronous bilateral breast carcinomas were more likely to undergo bilateral mastectomy, in which the breast-conserving rate was only 33% [10]. Furthermore, multicentric disease in the ipsilateral breast is basically contraindication to BCS. Careful and close follow-up are mandatory for this patient.

The case of bilateral breast carcinomas treated with bilateral volume replacement technique with the OF is presented. This unique technique can be considered in a highly selected patient.

## Conclusions

The OF can be considered for highly selected patients with bilateral breast carcinomas.

## Data Availability

Not applicable.

## Abbreviations

BCS: Breast-conserving surgery
OF: Omental flap
SNB: Sentinel lymph-node biopsy
IMF: Inframammary fold
